# Development of multifunctional unmanned aerial vehicles versus ground seeding and outplanting: What is more effective for improving the growth and quality of rice culture?

**DOI:** 10.3389/fpls.2022.953753

**Published:** 2022-07-28

**Authors:** Peng Qi, Zhichong Wang, Changling Wang, Lin Xu, Xiaoming Jia, Yang Zhang, Shubo Wang, Leng Han, Tian Li, Bo Chen, Chunyu Li, Changjun Mei, Yayun Pan, Wei Zhang, Joachim Müller, Yajia Liu, Xiongkui He

**Affiliations:** ^1^College of Science, China Agricultural University, Beijing, China; ^2^Centre for Chemicals Application Technology, China Agricultural University, Beijing, China; ^3^College of Agricultural Unmanned System, China Agricultural University, Beijing, China; ^4^Tropics and Subtropics Group, Institute of Agricultural Engineering, University of Hohenheim, Stuttgart, Germany; ^5^Yuren UAV (Zhuhai) Co., Ltd., Zhuhai, China; ^6^Agricultural and Rural Bureau of Huaiyuan, Huaiyuan, China; ^7^Anhui Difa Agricultural Technology Co., Ltd., Huaiyuan, China; ^8^Anhui Zhongke Intelligent Sense Technology Co., Ltd., Wuhu, China

**Keywords:** multifunctional UAV, seeding, fertilizing, plant protection, mechanization, CFD simulation, rice

## Abstract

The agronomic processes are complex in rice production. The mechanization efficiency is low in seeding, fertilization, and pesticide application, which is labor-intensive and time-consuming. Currently, many kinds of research focus on the single operation of UAVs on rice, but there is a paucity of comprehensive applications for the whole process of seeding, fertilization, and pesticide application. Based on the previous research synthetically, a multifunctional unmanned aerial vehicle (mUAV) was designed for rice planting management based on the intelligent operation platform, which realized three functions of seeding, fertilizer spreading, and pesticide application on the same flight platform. Computational fluid dynamics (CFD) simulations were used for machine design. Field trials were used to measure operating parameters. Finally, a comparative experimental analysis of the whole process was conducted by comparing the cultivation patterns of mUAV seeding (T1) with mechanical rice direct seeder (T2), and mechanical rice transplanter (T3). The comprehensive benefit of different rice management processes was evaluated. The results showed that the downwash wind field of the mUAV fluctuated widely from 0 to 1.5 m, with the spreading height of 2.5 m, and the pesticide application height of 3 m, which meet the operational requirements. There was no significant difference in yield between T1, T2, and T3 test areas, while the differences in operational efficiency and input labor costs were large. In the sowing stage, T1 had obvious advantages since the working efficiency was 2.2 times higher than T2, and the labor cost was reduced by 68.5%. The advantages were more obvious compared to T3, the working efficiency was 4 times higher than in T3, and the labor cost was reduced by 82.5%. During the pesticide application, T1 still had an advantage, but it was not a significant increase in advantage relative to the seeding stage, in which operating efficiency increased by 1.3 times and labor costs were reduced by 25%. However, the fertilization of T1 was not advantageous due to load and other limitations. Compared to T2 and T3, operational efficiency was reduced by 80% and labor costs increased by 14.3%. It is hoped that this research will provide new equipment for rice cultivation patterns in different environments, while improving rice mechanization, reducing labor inputs, and lowering costs.

## Introduction

Rice is one of the most important crops throughout the world. As a staple food for more than half of the world’s population, rice is cultivated in more than 100 countries, and 90% is produced in Asia (Bhandari, 2019; Fukagawa and Ziska, 2019). The rice cultivation area in China is about 30 million hm^2^, accounting for 30% of the crop cultivation area in China and 20% of the rice cultivation area around the world. The total rice production is nearly 20 million tons in China, accounting for 40 of total grain production in China and 35% of the total rice production all over the world (Zhu et al., 2007; He et al., 2008). With 60% of the Chinese population relying on rice as a staple food, rice has the largest area of cultivation, and the highest yield, which occupies an extremely important position in food security. (Feng et al., 2020).

However, there are many segments and complex agronomic technical measures in rice cultivation, which lead to the low-efficiency mechanization of the whole production process. There are great differences in the mechanization levels of rice seedling, transplanting, fertilization and pesticides management, machine harvest, postharvest transportation, and grain drying (Chen et al., 2017b). The rice cultivation process is divided into four main parts: tillage, seeding, management, and harvest. The tillage and harvest are mechanized, but the seeding and management still cannot meet the requirements of modern agriculture (Li et al., 2012; Song et al., 2018b). Especially in the hilly areas, the arable land is small and scattered, where the terrain is rugged. There are also problems with the use of mechanical operations on the plains. For example, the soil is compacted resulting in uneven seed emergence, and there is an inconvenience in the use of small farm machinery (Lan et al., 2017). They are time-consuming, inconvenient, and labor-intensive, which seriously affect the progress of mechanization (Bao and Li, 2004; Zhang and Zhou, 2019).

With the development of modern manufacturing, many forms of agricultural equipment are used in rice production. Unmanned aerial vehicle (UAVs), with the advantages of fewer site requirements, low energy consumption, high safety, and no space restrictions, has been widely used in rice agricultural production (Wang et al., 2019). The continuous improvement of agronomic technology, intelligent supporting technology, and equipment for the whole process of rice production have been introduced, which promoted many scholars to use UAV applications for rice management (Zhang and Gong, 2014; Zhou et al., 2017, 2019). Firstly, for the effect of plant protection UAV operating parameters on droplets and control effectiveness, some previous explorations have been conducted. Wang et al. (2017) studied the effect of spray parameters of small, unmanned helicopters on the deposition of droplets. The experimental results showed that the droplet distribution decreased from the upper to the lower layer of the rice canopy and decreased with the increase of flight speed. Chen et al. (2016) studied the effect of HY-B-10L unmanned helicopter spray parameters on the droplet deposition distribution in the hybrid rice canopy. The results showed that the vertical wind field above the plant canopy weakened with increasing height, and the amount of droplet deposition gradually decreased; the lower the operating speed, the more droplets were deposited below the aircraft. Xue et al. (2013) applied N-3 UAV to investigate the effect of different flight heights on the control of rice *Planthopper* and *Cnaphalocrocis medinalis*. The results showed that low flight altitude was effective in controlling rice *Planthopper* and *C. medinalis*, and the application volume was directly proportional to the control effect at the same flight altitude. Secondly, for agricultural UAV seeding, our researchers have conducted a lot of research. In 2014, Li et al. (2016) conducted UAV spreading experiments. The results showed that a 0.09 hm^2^ rice field only required 305 s to complete rice seed spreading. Cheng and Li (2020) studied the effects of direct seeding on rice growth characteristics and yield. The results showed that UAV direct seeding had significant advantages over hole-direct seeding and manual sowing in seedling quality indexes. The yield of UAV direct seeding was 454.9 kg/667 m^2^, which was higher than that of manual sowing at 417.9 kg/667 m^2^, but lower than that of hole direct seeding at 509.3 kg/667 m^2^. Thirdly, UAVs had also been studied for agricultural fertilization. Ren et al. (2021) designed a rice fertilizer spreading system. The test results showed that fertilizer spreading uniformity was significantly influenced by flow rate, UAV flight speed, centrifugal disc speed, and drop-in position angle, all of which interacted with each other. The best fertilizer spreading performance was achieved when the drop-in position angle was forty, the centrifugal disc speed was 1,100 r/min, the fertilizer flow rate was 3,460 particles/s, and the flight speed was 5 m/s. The coefficient of variation was 8.86% currently. The fertilizer application efficiency of the UAV was about 12.5 times that of manual fertilizer application.

In 2012, there were less than 10 Chinese agricultural UAV manufacturers and only a few hundred agricultural UAVs. With the promulgation of the Centra Document No. 1 in 2014, the number of UAV enterprises have increased by nearly hundreds, and the sales amount has reached 60 thousand units by 2020. Correspondingly, agricultural UAV ownership has reached 110 thousand units (Yubin and Guobin, 2018; Yongwang et al., 2020). The rapid development of the UAV industry and the rise in labor costs have accelerated the use of UAVs in the rice cultivation process. Especially in areas where large ground machinery cannot operate rice cultivation, the application of UAVs has broad prospects.

**FIGURE 1:**
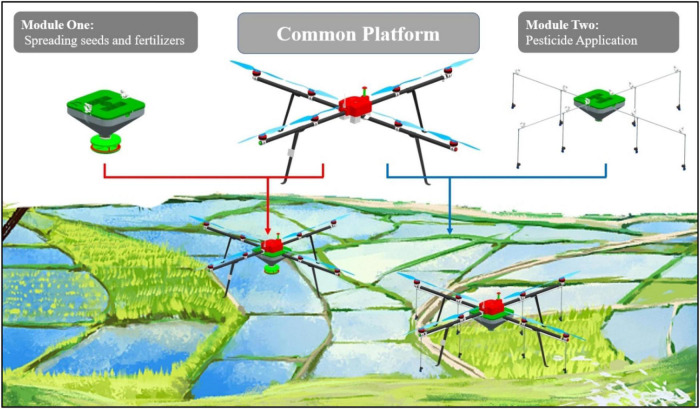
mUAV total design solution.

Currently, UAVs are widely used in different aspects of agriculture, but mostly single function. UAVs have developed rapidly in agricultural fields, such as disease and insect pest control, pollination, and agricultural information acquisition by remote sensing, but the development of UAV air sowing is relatively backward (Wan et al., 2021). Considering the characteristics of different application of UAVs in seeding, fertilization, and pesticide application in rice cultivation, the idea to design an multifunctional unmanned aerial vehicle (mUAV) is proposed (Figure 1), which can combine all these functions. The focus of this article mainly included the following aspects: (1) The mUAV were structured as a modularity of different functions. A sophisticated flight platform was designed and improved, which was based on modular design theory connected to different functional devices, and these different modules corresponded to different functions. This structure could increase the utilization rate of machinery and reduce the idle rate of agricultural machinery. It could also reduce the acquisition cost of agricultural machinery and protect farmers’ income. (2) The appropriate range of operational parameters was selected for the different functional modular devices. Two approaches were used to evaluate the operational patterns separately. Numerical simulation analysis was prioritized, which focused on the downwash airflow motion regulation. Afterwards, field tests were combined for verification, which clarified the particle distribution at the target under the action of downwash airflow. It was hoped that the content of these studies would provide some guidance for practical use by farmers. (3) Comparative field trials were conducted in the mUAV, mechanical seeding, and mechanical transplanting the latter two, of which were the mainstream mechanized operations in society. Each of the three types of equipment was applied to the whole process of rice cultivation to summarize and analyze the impact of labor cost, as well as rice yield in the whole process of rice cultivation. It was hoped that the comparison test would provide a reference basis for farmers to recognize and understand the advantages and weaknesses of the mUAV when they chose the mUAV for their operations.

## Materials and methods

### Multi-rotor unmanned aerial vehicle platform and multifunctional component design

**FIGURE 2:**
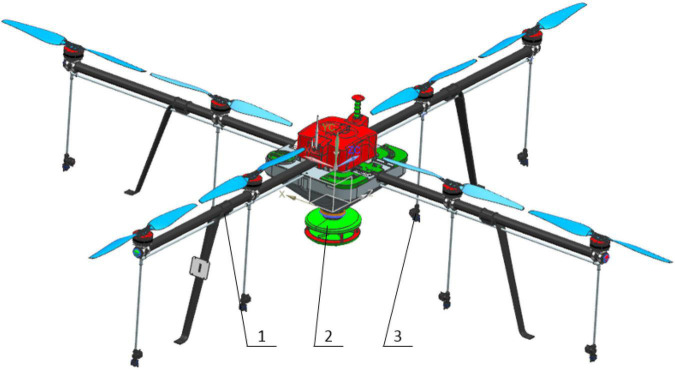
3D overall design of mUAV. 1 represents the public flight control platform, 2 represents the device for spreading seed or fertilizer, and 3 represents the entire system used for pesticide application.

In this research, based on the current UAV models, an eight-rotor UAV with 20 kg loading capacity was designed based on “Yuren” automatic flight control system. Multifunctional component system was designed based on this UAV platform. Its core working components were mainly divided into three parts: flight control platform, spreading system, and spraying system. The spreading system and the spraying system were connected with the rotary arm through the quick release buckle to realize the agricultural various agricultural operation requirements of sowing, spreading fertilizer, and spraying. A three-dimensional model of the whole structure was constructed using Unigraphics NX (Siemens PLM Software), as shown in Figure 2. The basic parameters were listed in Table 1.

**TABLE 1:** Basic parameters of the multifunctional UAV.

Parameters	Values
Dimensions/mm^3^	3,740 × 3,740 × 800
Fight velocity/ms^–1^	0–7
Fight altitude/m	0.5–5
Number of nozzles	8
Number of rotors	8
Spaying width/m	8–10
Spreading fertilizer width/m	9–12
Seeding width/m	5–7
Maximum load/kg	20
Battery capacity/mA⋅h	5,500

### Operational parameters optimization

#### Multifunctional unmanned aerial vehicle wind field computational fluid dynamics simulation by using ANSYS

The wind field below the UAV rotor was mainly composed of the UAV rotor wind field and the external environment wind field, which was the main factor affecting the particle trajectory. During the stable flight of the UAV, a strong rotor wind field would be generated, which had a certain coercive effect on the particle movement. Therefore, the computational fluid dynamics (CFD) method was used to explore the influence of the mUAV rotor wind field on the particle movement. Through the research on the distribution characteristics and development regularity of the mUAV rotor wind field, the influence of the mUAV rotor wind field on the particle movement can be more accurately explored and understood.

**FIGURE 3:**
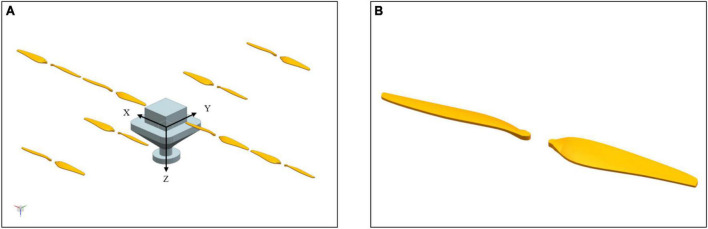
The simplified structure for numerical simulation processes. **(A)** Represents the simplified rotor and other structures, while **(B)** represents the specific structure of each propeller after simplification.

An accurate 3D model of mUAV played a key factor in CFD simulation, but the complex structure led to difficulties in modeling and boundary conditions. As shown in Figure 2, the mUAV is a full-size structure, which was not necessary because the shape of the mUAV was a complex-curved surface. Therefore, simplifying the structure was very necessary for CFD simulation. The simplified result was shown in Figure 3.

In the 3D coordinates, the forward motion in *X* direction was the front of the MUVA flight, the forward motion in *Y* direction was the right offset, and the forward motion in *Z* direction was the descent direction.

**FIGURE 4:**
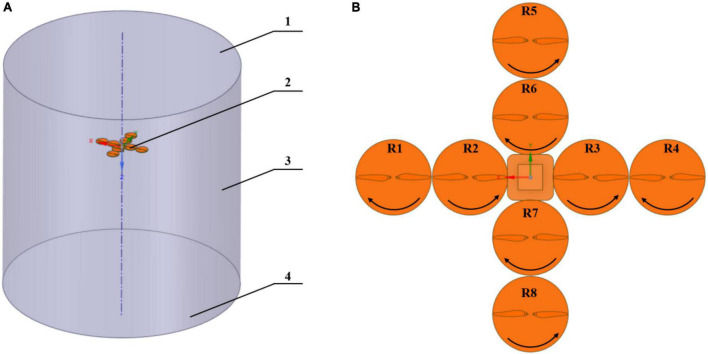
Computational domain settings in numerical simulation. **(A)** Stands for the name of the boundary condition, where 1 represents the top surface of the computational domain, 2 represents the location of the mUAV, 3 represents the space wall, and 4 represents the ground. **(B)** Stands the top view of the rotor in the computational domain, where R1–R8 represents each rotor, respectively, and the corresponding black portion of arrows represent the direction of rotation of that rotor in the computational domain.

The mUAV was located at the center of the coordinate system (*X* = 0, *Y* = 0, *Z* = 0) and the distances between the mUAV and the ground were 1–6 m. The entire computational domain was a cylinder with a radius of 8 m, as shown in Figure 4A. The rotation direction of the mUAV blades was shown in Figure 4B. The eight rotors of the mUAV were the rotational domain and the cylindrical air was the stationary domain. The interface meshes were established for accurate calculation between rotors domain and air domain.

The SpaceClaim software (Release 2021 R1, Ansys, Inc., PA, United States) was used to develop the CFD models of simplified mUAV model, Mesh in Workbench was used for meshing and as the solver. The unstructured tetrahedral mesh was applied, and the size of the grids was defined. The mesh size of the rotational domain was defined as 10 mm, the mesh of the stationary domain was defined as 400 mm, and the mesh quality was improved by defining the dimensions of faces and lines separately. Also, it had turn on features, such as Capture Curvature and Proximity to improve mesh quality. Transient SST k-ε model was employed (Wang et al., 2001; Omar et al., 2016), the computation lasted 1,000 steps with a time step of 0.005 s. The workstation was used to calculate the results of the rotational speed of the rotating domain at 2,500 r/min.

#### Experimental parameters

Seeds and fertilizers were evenly distributed in the soil, which was beneficial to seed emergence and balanced fertilizer nutrition. Therefore, refer to the test regulations of the technical specifications in EN13739-1 (2011) Agricultural machinery – Solid fertilizer broadcasters and full width distributors – Environmental protection and NY/T1003 (2006) Technical specifications of quality evaluation for fertilizing machinery, the distribution of particles in the field was an important test parameter. To reduce the particle bouncing and other factors affecting the collection efficiency, particle size uniformity detector (AAMS-SALVARANI BVBA, Germany) was used as a dedicated tester to collect particles. During the test, Pocketwind IV Anemometer (Lechler GmbH⋅Agricultural Nozzles and Accessories, Germany) was used as a device for recording wind speed and temperature of the environment. If the wind speed exceeded 4 m/s and the temperature exceeded 30°C, it would be regarded as invalid data. In the experiment, about 667 m^2^ of open space was selected as the UAV operation area, and the center of the area was used as the collection location of the detector. When the UAV was operating, the detector area would be fully covered to ensure accurate data. The speed of the UAV was set between 2 and 2.5 m/s and keep the flight height at 1.5, 2, and 2.5 m, respectively. After the UAV landed safely, the particles in each detector were recorded.

**FIGURE 5:**
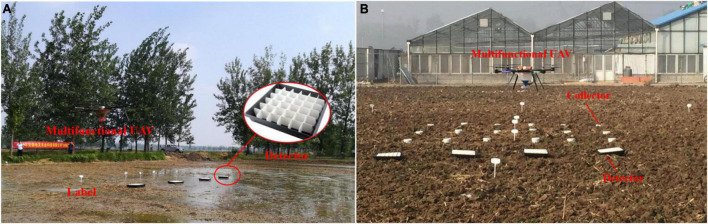
Test site for rice seeding and fertilization. **(A)** Represents the rice seeding test site, where detector is to collect the rice seeds that fall on the ground; Label is to identify the location. **(B)** Represents the fertilizer spreading test site, where detector is used to collect ground fertilizer particles, and collector is used as another way to collect ground fertilizer particles.

For the sowing test (Figure 5A), to simulate the real working environment, the fields, which were soaked in water, were selected. The detectors were fixed in the test field with an interval of 2 m and perpendicular to the UAV course. The detector was divided into 6 × 6 partitions, with an outer size of 500 mm × 500 mm, which can easily count the number of seeds in each small partitions. There was some variation in the location of fertilizer test site (Figure 5B), which was selected on a dryland. In addition to using a collector, Φ200 mm × 500 mm size circular collectors were used to collect fertilizer particles in a 3 × 6 array at 1,500 mm × 1,200 mm intervals.

**FIGURE 6:**
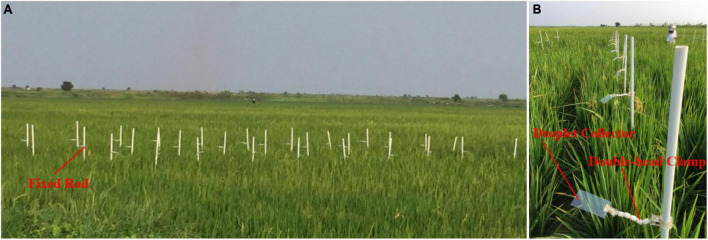
Rice pesticide application test site. **(A)** Represents the field sampling site of rice field, where the sampling points are arranged on the fixed rod. **(B)** Represents a partial schematic of each fixed rod, where the double-head clamp connects the fixed rod to the droplet collector.

The plant protection application experiment was conducted at the jointing-booting stage of paddy. The fixed rods were inserted into the paddy field, which were arranged in a 3 × 9 matrix with a cortege spacing of 3,000 mm × 1,000 mm (Figure 6A). One end of the double-head clamp was fixed on the fixed rod, and the other end clamped the droplet collector (Polyethylene Card, PVC), as shown in Figure 6B. The two droplet collectors were located at the top and middle ends of the same fixed rod. It was placed horizontally, which accounted for 100 and 50% of the length of the rice, respectively. The Allura red solution with a concentration of 10 g/L was prepared as a droplet deposition tracer, which was put into the UAV tank before flight. The UAV was operated with the parameters shown in Table 2. The Wind Master model (Gill Instruments Ltd., United Kingdom) was used on site to monitor the environmental parameters. After the UV landed, the sampling bottle collected 50 ml of liquid in the tank. After the droplet collectors were naturally dried, each droplet collector was individually packaged in a plastic sealing bag and stored in darkness.

**TABLE 2:** Spray test and environmental parameters.

Test number	Flight height (m)	Flight speed (m⋅s^–1^)	Wind speed (m⋅s^–1^)	Temperature (°C)
1	1.0	6.0	2.7	34.6
2	2.0	6.0	2.5	32.5
3	3.0	6.0	2.2	31.2
4	3.0	4.0	2.6	30.4
5	3.0	5.0	2.7	32.7
6	4.0	6.0	1.7	30.5

Refer to ISO22866 (2005) and ISO24253-1 (2015) standards. Deionized water (50 ml) was added to the plastic sealing bag of polyethylene film, then the plastic sealing bag was placed in a shaker at 500 r/min for 10 min. The eluate was measured with a 722 spectrophotometer. The droplet deposition refers to the mass of the droplet per unit area, which was calculated using the following Eq. 1 (Qin et al., 2014; Wang et al., 2020):


(1) $$\beta_{\mathrm{dep}}=\frac{\left(\rho_{s\,m\,p\,l}-\rho_{b\,l\,k}\right)\times F_{e\,a\,l}\times V_{d\,i\,i}}{\rho_{s\,p\,r\,a\,y}\times A_{c\,o\,l}}$$

β_*dep*_: Droplet deposition [$\frac{\mu \,g}{c\,m^{2}}$]; ρ*_smpl_*: Sample Collection Values; ρ*_blk_*: Blank Control Values; *F*_*eal*_: Calibration Factor (per fluorimeter scale unit) [$\frac{\mu \,g}{L}$]; *V_dii_*: Volume of dilution liquid used to solute tracer from collector [L]; ρ*_spray_*: Tracer concentration in spray liquid [$\frac{\mu \,g}{L}$]; *A_col_*: the area of the collector for catching the spray deposition[*cm*^2^].

The uniformity of the particles or spray deposition on the sampling places are reported as the coefficient of variation (CV) of the measured deposition values, and a lower CV value indicates a better uniformity of deposition distribution. Equation 2 describes the calculation of the CV (%) using the ISO standard 24253-1.


(2) $$\mathrm{CV}=\frac{\sqrt{\sum_{i=1}^{n}\frac{{\left(X_{i}-\overset{-}{X}\right)}^{2}}{\left(n-1\right)}}}{\overset{-}{X}}\times 100\%$$

*X*_*i*_: Samples from each collection point; $\overset{-}{X}$: Corresponding to the average value of the collected samples; *n*: Number of samples collected.

All statistical analyses were performed using IBM SPSS Statistics for Windows (IBM Corp., Armonk, NY, United States). Two-way analysis of variance (ANOVA) was adopted to explore the effects of broadcast and spray on distribution uniformity on the field. In all trials, the mean values of distribution uniformity at different parameters, together with those of percentage, were compared using one-way ANOVA via the Duncan test (α = 0.05).

### Application experiment of rice cycle cultivation management

To better understand the performance of mUAV operated in an outdoor environment, comprehensive comparison experiments were conducted at different growth stages of rice using different agricultural machinery.

There were three main modes of local rice cultivation: The first one was rice direct seeder cultivation, which was an important way to fully mechanize rice cultivation. The second one was mechanized rice transplanter, which is also an important mode and guarantees and supports mechanization planting. The third was artificial transplanting, which is a traditional rice transfer method that had been gradually replaced by mechanical transplanting. Therefore, the comprehensive comparative experiment was set up with three cultivation patterns: the first experimental field was the multifunctional UAV directly sowing, fertilizing, and applying pesticide. The second experimental field was sown with a rice direct seeder, and fertilizer and pesticide were applied using other ground machinery. The third experimental field was sown with a rice transplanter, while other ground machinery was used to apply fertilizer and pesticides. The area of each experiment area was 2.2 hm^2^, which were marked as T1, T2, and T3 respectively.

Full mechanization referred to the mechanization of efficient production technologies in rice production, such as tillage, planting, plant protection, fertilization, harvesting, drying, and straw treatment. According to the agricultural characteristics and experimental requirements of rice cultivation, there were five main segments: tillage, seeding, planting, fertilization, and harvesting. This experiment was conducted to compare the feasibility of UAV application in the whole process of rice and a comprehensive comparison with two mechanical planting methods. The test site was in Zhuangqiao Village, Wanfu Town, Huaiyuan County, Bengbu City, Anhui Province in 2020, and the trial included evaluation of labor costs, rice yield and profit, cost analysis of transplanting, etc. To match the actual production conditions, the agricultural materials and equipment used during the experiments process were provided by local cooperatives.

#### Agronomic process of different cultivation modes of rice

##### Ploughing stage

**FIGURE 7:**
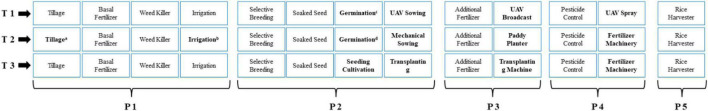
Agricultural process of rice cultivation in different experimental areas. *^a^* Compared to T1 and T3, the workload in the T2 region is reduced by about half, *^b^* compared to T1 and T3, the T2 area is only about 60% irrigated, keeping the soil from drying out, but not having standing water, *^c^* the seeds germinated to 2 mm, which took about 20 h, *^d^* the seeds were just ready to germinate, but did not grow, taking about 8 h. T1 represents mUAV experiment area, T2 represents mechanical rice direct seeder area, T3 represents mechanical rice transplanter area. P1 represents soil preparation stage, P2 represents seeding stage, P3 represents fertilization stage, P4 represents pesticide application, and P5 represents harvester stage.

All experimental fields were cultivated with a 1804D model tractor (YTO GROUP CORPORATION, China) and a 1GS-3300 model rotary tiller (YTO GROUP CORPORATION, China) with a width of 3,000 mm for the purpose of breaking the soil stubble. At the same time, 40 kg/667 m^2^ base fertilizer was applied, and the soil and fertilizer were mixed using rotary tillage. In the field, a ditch with a depth and width of 300 mm × 300 mm was dug at an interval of 4,000 mm for water retention and to prevent waterlogging. The herbicide was sprayed by a 3WPZ-1500B self-propelled boom sprayer (Qingzhou Aike Machinery Technology Co., Ltd., Qingzhou, China). The difference was that T1 and T3 irrigations were kept for 7 days, and the water was drained, while T2 did not require further treatment. As shown in Figure 7.

##### Sowing stage

In the T1 experimental area, the seeds were soaked 24 h in advance and germinated to a length of 1.5–2 mm (Tao et al., 2011), and the seeds were sown into soil at 5 kg/667 m^2^ using a UAV broadcast. In the T2 experimental area, the seeds were only soaked about 12 h until just about to germinate, and the seeds sown into soil at 6 kg/667 m^2^ using a rice direct seeder (Huaiyuan County Sanliu Agricultural Machinery Co., Ltd., Huaiyuan, China). In the T3 experimental area, the seeds were cultivated 20 days in advance, which was the seedling raising period, and then the seedlings were collected and transported, then 4–7 seedlings per hole were inserted into the experimental field with a row spacing of 140 mm × 300 mm using a NSD8 model mechanical rice transplanter (Kubota Agricultural Machinery (Suzhou) Co., Ltd., Suzhou, China). According to the agronomic characteristics, T1 and T2 were selected as suitable for direct seeding with the same seed variety, and T3 was selected as another variety suitable for transplanting.

##### Top dressing stage

Topdressing was mainly in the tillering and booting period, and the amount of fertilization was 15 kg/667 m^2^ each time, for a total of two times. The main difference between the three experimental areas were the use of different equipment. In the T1 area, the feeding port suitable for the caliber was installed to the UAV broadcast, and then the top-dressing operation was carried out. In the T2 and T3 experimental areas, to reduce the cost of agricultural equipment, the sports chassis of the 3WPZ-1500B self-propelled machine, and the separately purchased fertilizer spreader were used to work together.

##### Pesticide application stage

In the actual management of diseases and pests, pest prevention is the main method of pest and disease control. Therefore, according to the needs of agricultural control and the occurrence of pests and diseases, pesticides were generally sprayed before the occurrence of pests and diseases. The pesticides were mainly insecticides and fungicides, which were sprayed 3–4 times during the rice cultivation process. The main differences between the three experimental fields were the agricultural equipment and the amount of pesticide applied. In the T1 experimental area, the application system was installed on the UAV platform, and the pesticides were mixed and diluted with an application rate of 1.5−2 L/667 m^2^. The 3WPZ-1500B self-propelled boom sprayer was used in the T2 and T3 experimental areas to spray with the application rate of 15−20 L/667 m^2^.

##### Harvest stage

The rice yield was evaluated, reference to the National Grain High Yield Creation Yield Measurement, Acceptance Method, and the DB32/T 1093-2015 Standard. Five units were selected in the diagonal direction of each experimental area, each unit of 20 × 667 m^2^ was used as a production measurement unit. Five sampling points were randomly selected in each production measurement unit. For the experimental field with obvious row spacing and plant spacing, representative rice panicles within a certain range were selected from each sampling point, and the number of panicles per 667 m^2^ was calculated. Representative mature rice ears with ten holes per unit were selected to measure the plant height and root length of rice. After natural air-drying, the number of grains per ear, seed setting rate, and thousand-grain weight were determined, and the theoretical yield per 667 m^2^ was calculated. For the experimental field where the row spacing and plant spacing cannot be clearly distinguished, a square frame of 1,000 mm × 1,000 mm was made with hard iron wire, and the number of ears and yield per 667 m^2^ were calculated. Finally, the 4LZ-200 rice combine harvester (Luoyang Zhongshou Machinery Equipment Co., Ltd., Luoyang, China) was used to harvest rice.

## Results

### Characteristics of downwash airflow at different heights

**FIGURE 8:**
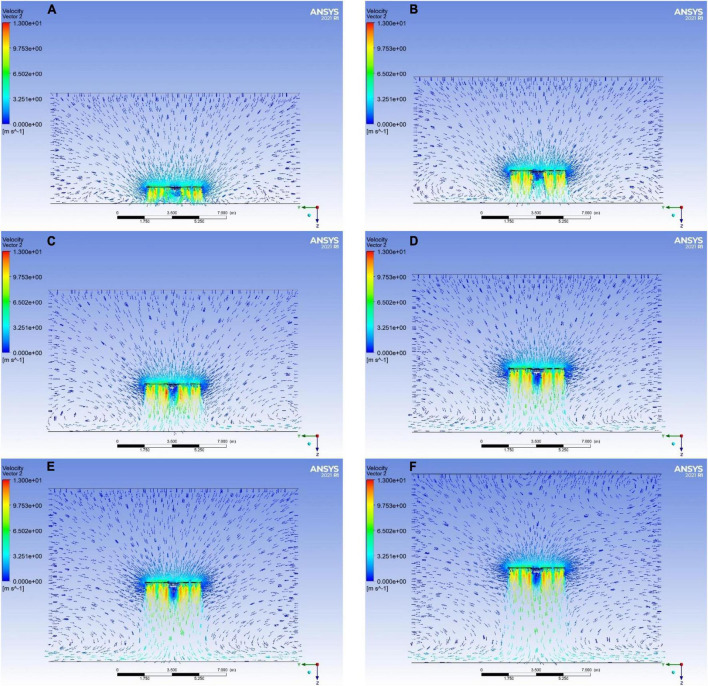
Flow field velocity vector illustration of the mUAV flying at different altitudes. **(A–F)** Indicate that the mUAV is flying at 1, 2, 3, 4, 5, and 6 m above the ground, respectively.

As shown in Figure 8, the velocity of the downwash flow field was varied along the *Z*-axis in the range of 1–6 m from the ground for the UAV.

Higher wind speeds were generated at the wingtips of the rotor blades, and two distinct areas of acceleration appeared below each rotor blade. An upward (*Z*-axis negative direction) airflow appeared in the center of the mUAV broadcast. The vertically downward scrubbing airflow created a negative pressure that caused the surrounding air to converge on the downward scrubbing airflow. The vertically downward airflow touched the ground, causing the airflow to flow sideways, and this airflow met the airflow that was brought to the center, forming a vortex.

At the same time, various combinations of altitudes can be seen. As the flight height of the mUAV increased, the velocity change tended to level off at the position near the ground, the airflow gradually increased along the radius direction, and the vortex gradually moved away from the center.

**FIGURE 9:**
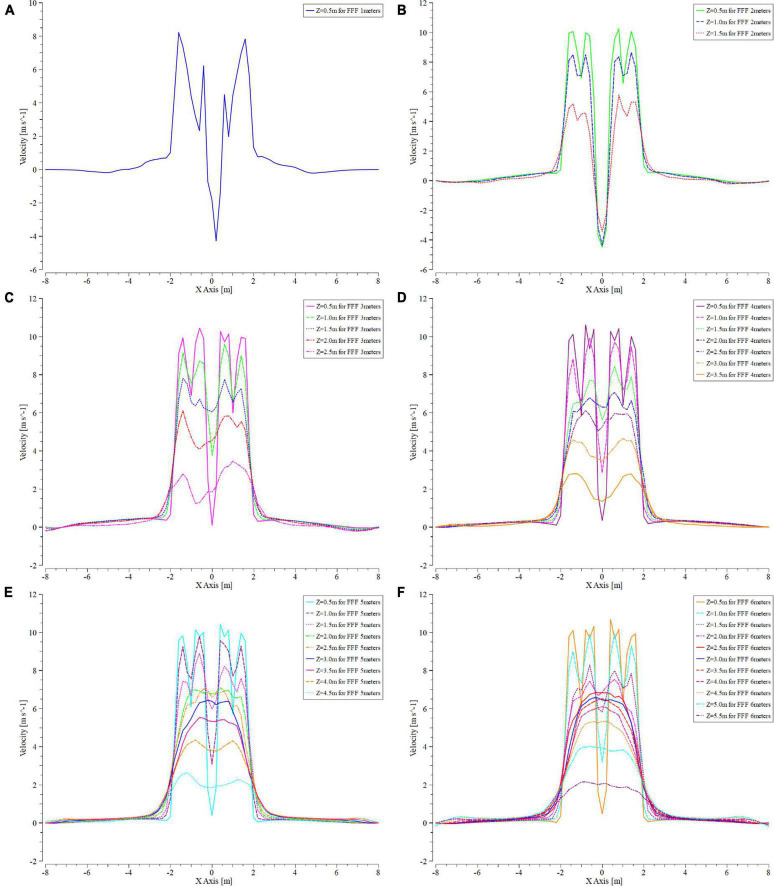
Variation curve of velocity at different altitudes during simulation. **(A–F)** Indicate that the mUAV is flying at 1, 2, 3, 4, 5, and 6 m above the ground, respectively. The “Z” in each figure indicates the distance from below the mUAV, “for FFF meters” indicates the altitude at which the mUAV is flying. The curves in each graph indicate the wind speed of the mUAV at distance “Z,” where positive values indicate a vertical downward wash and negative values indicate a vertical upward wash.

In all the interfaces shown in Figure 8, a straight line was set at 0.5 m intervals along the *Z*-axis direction. This straight line indicated that the velocity changed at different distances in the *Y*-axis (Figure 9).

Four small peaks and one trough were observed at 0.5 m (Figure 9A) and 1 m (Figure 9B) below the UAV at all test heights. The wind speed at this point was unstable and there was an effect of wind speed in the opposite direction (*Z*-axis negative direction). Since the mUAV was so low to the ground, the strong airflow hit the ground and bounced off the ground, which referred as the ground effect.

An upward (*Z*-axis negative direction) velocity was observed in the range of 0.5–1.5 below the mUAV at flight heights of 1 and 2 m. Until the flight altitude of 3 m (Figure 9C), the velocity direction was still downward (positive *Z*-axis direction) despite the sharp change in the center of the mUAV. Moreover, above 1.5 m from the bottom of the mUAV, the velocity change tended to smooth out.

In addition, the flow field below the mUAV became more stable with the increasing flight altitude, and the same trend was observed at flight altitudes of 4–6 m (Figures 9D–F).

According to the above simulation results, it was suggested that the mUAV should operate at a height of 2 m or more.

### Variation of spread distribution with height

**FIGURE 10:**
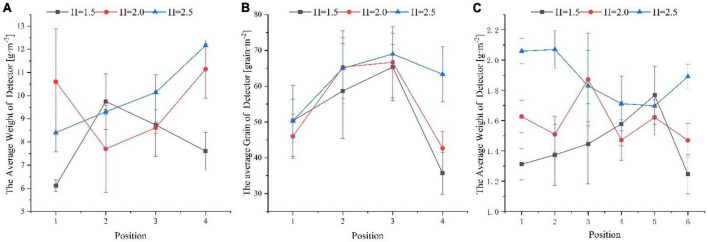
The uniformity of spreading in the horizontal direction at different heights. **(A)** Represents uniformity of horizontal distribution of sown seeds. **(B)** Represents uniformity of horizontal distribution of fertilizer in square collectors. **(C)** Represents uniformity of horizontal distribution of fertilizer in circular collectors. In all figures, the horizontal coordinates indicate the collector number, and the vertical coordinates indicate the distribution, where “h” indicates the flight height of the mUAV from the ground.

When the mUAV was used for seeding tests, the flight altitude had an (positive or negative) effect on the uniformity of spreading, which was 24.36, 22.83, and 13.05% in the horizontal direction for the three altitudes tested. As shown in Figure 10A, the fluctuations were relatively large at the position of test point (1,4) and less in the middle (2,3).

When the mUAV performed fertilizer spreading was tested, there was the same trend at different heights, and the weight of particles collected by the detector that showed more in the middle and less on both sides. The variability was greater on the right side than on the left side, as shown in Figure 10B. In the circular detector (Figure 10C), the left collector had a large particle dispersion and good uniformity of dispersal to the off-right of the mUAV route. The CV in the horizontal direction fluctuated from 11.98 to 23.68% over the range of test heights.

According to the simulation results (Figure 9), the mUAV tended to have a smoother downwash airflow with an increasing height, which was more conducive to the uniform distribution of particles. However, the actual motion of the particles in the air varied greatly under the influence of the ambient wind field forces. The higher the flight height, the longer the fall time in the air and the more uncontrolled the trajectory of the particles, which led to greater uncertainty in the particle fall point. As shown above, 2.5 m would not be the most suitable height, but it could meet the requirements of agronomic spreading and minimize the influence of ambient wind field forces.

### Variation of spray distribution with height and velocity

**FIGURE 11:**
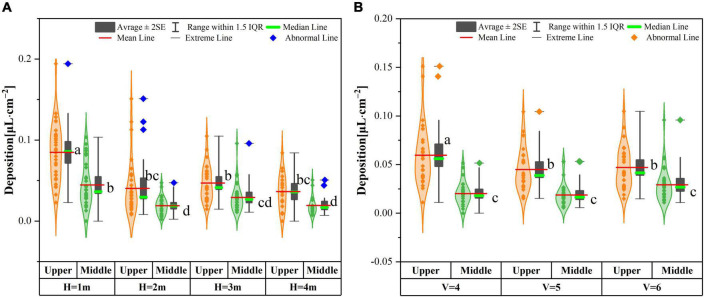
Distribution characteristics of droplet deposition in the rice canopy. **(A)** Represents deposition distribution affected by flight heights, **(B)** Represents deposition distribution at affected by flight speeds. Where “H” represents the mUAV flight height, “V” represents the mUAV flight speed, “Upper” represents the upper rice layer, and “Middle” represents the middle rice layer. a, b, c, and d indicate a significant difference at *P* < 0.05.

As shown in Figure 11A, the deposition of droplets in the upper and middle of rice was affected by the flight height of the mUAV. The low flight (*H* = 1 m) can significantly increase the deposition of the upper and middle, and the deposition in the upper and middle layers was 0.0849 and 0.0446 μL cm^–2^. From *H* = 2 m to *H* = 3 m, there was no significant difference in the deposition between the upper and middle of rice, but the deposition at *H* = 3 m was higher than that at *H* = 2 m and *H* = 4 m. In the range of UAV flight speed of 4–6 m s^–1^ (Figure 11B), low flight speed could significantly increase the deposition of droplets in the upper layer, but it had no significant effect on the deposition of the middle.

**FIGURE 12:**
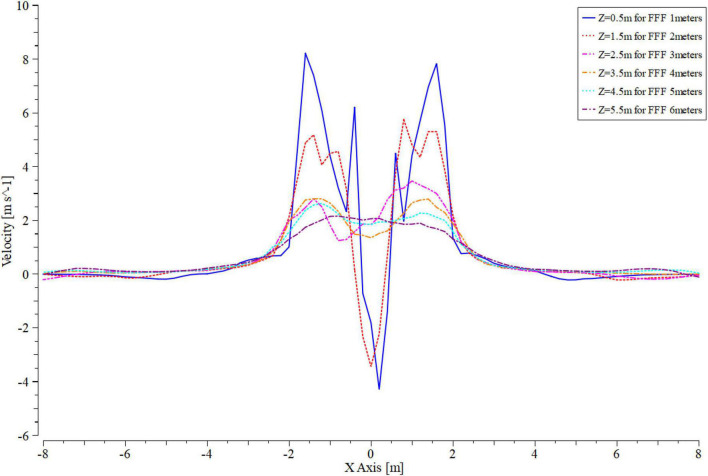
Velocity variation curve at 0.5 m from the ground at different flight heights. Where “*Z* = 0.5 m for 1 m,” “*Z* = 1.5 m for 2 m,” “*Z* = 2.5 m for 3 m,” “*Z* = 3.5 m for 4 m,” “*Z* = 4.5 m for 5 m,” “*Z* = 5.5 m for 6 m,” the size of the downwash airflow at 0.5 m from the ground when the UAV is flying at 1, 2, 3, 4, 5, and 6 m, respectively.

When the mUAV was flying at an altitude of 1–6 m above the ground, Figure 12 showed the wind speed profiles for different flight heights of 0.5 m above the ground. The intensity of the downwash airflow was highest and unstable when the mUAV was flying at 1-m height, which resulted in a high air mass flow rate and an increase in the number of particles per unit time of droplet movement, which eventually manifested itself as an increase in deposition. One possibility inferred from this was that the downwash airflow velocity of the mUAV above the canopy should be less than 5 m/s to evenly distribute the deposition above and below the canopy.

Under the same speed conditions, the droplet deposition in the upper and middle layers tended to decrease with the increase of flight height, which may be affected because of droplet drift caused by the increase of height. Under the condition of the same flight height, the deposition of the upper and middle also decreased with the increase of the speed. This was caused by the low flight speed, which led to the increase of the spraying flow droplet deposition. According to the CV values of the droplet’s deposition and distribution in the rice canopy, the 3-m flying height with a speed of 5 ms^–1^ were selected/recommended during the spraying operation.

### Yield of different cultivation patterns

After analysis of the yield components, it was found that the panicles distribution density, spikelet per panicle, as well as thousand grain weight, were all significantly affected by the methods of cultivation (Table 3). Meanwhile, the performance of T1 with T2 in the number of panicles per square was similar, while T3 had the lowest value (*P* < 0.05) among these methods. T1 and T2 were 19.7 and 9.5% higher than T3, respectively. The cultivation methods did not have an obvious difference of the number of spikelets per panicle. T2 increased the value (*P* < 0.05) by 24.6% compared with T3. The thousand grain weight were similar between T1 and T2, approximately 14.6% less in T1 compared with T3 area. However, there were no significant differences in seed-setting rate and predicted yield among the three experimental areas.

**TABLE 3:** Effect of different cultivation methods on grain yield and its components of rice.

Test area	No. of panicle per square (No/m^2^)	No. of spikelet per panicle	Seed-setting rate (%)	Thousand grain weight (g)	Yield (kg/667 m^2)^
T1	374.83a	121.93ab	94.52a	22.57b	577.41a
T2	342.83ab	140.37a	94.80a	23.50b	600.99a
T3	313.17b	112.67b	95.57a	26.42a	554.43a

a, b indicates a significant difference at P < 0.05.

### Comparison of benefits of different cultivation methods

The experimental site in this study was professionally managed by a cooperative production, and the statistics were based on workers employed and agricultural equipment purchased by the company.

As shown in the Table 4, the T1 area (mUAV broadcast) had significant advantages in the seeding session. Its operational efficiency was 2.2 times and 4 times higher than T2 and T3, respectively. The effect of pesticide spraying in T1 area was also higher than T2 and T3 area by 33%. However, the fertilizer application efficiency in T1 was five times lower than T2 and T3.

**TABLE 4:** Efficiency and number of employees of sowing, fertilizing, and application operations in different experimental areas.

Test area	Agronomic sessions	Work efficiency (667 m^2^/day)	Labor cost (RMB/day)	Number of employees/day	Number of operations*
T1	Sowing	200	350	2	1
	Fertilization	160	350	2	2
	Spraying	400	350	2	3
T2	Sowing	90	500	3	1
	Fertilization	800	1,500	4	2
	Spraying	300	350	2	3
T3	Sowing	50	500	3	1
	Fertilization	800	1,500	4	2
	Spraying	300	350	2	3

* Number of operations throughout the growth cycle of rice.

According to the management company salary standard, the driver should be paid RMB 200 per day, and the support staff should be paid RMB 150 per day. All agronomic segments required one driver and several support staff. The number of employees used for planting was the regular operation configuration. The seeding segment in T1 area had one less support staff than T2 and T3 areas, and the fertilizer stage had two less support staff than T2 and T3 areas.

As shown in Figure 7 in the process of P1 to P5, rice harvesting cost (P5) was the same and the difference in labor cost was mainly in P1 to P4. The labor cost was calculated based on the number of employees, the number of operations, and the work efficiency (Table 5).

**TABLE 5:** Labor cost of soil preparation, sowing, fertilizing, and spraying in different experimental areas*.

Test area	Soil preparation cost**/ RMB	Sowing cost/RMB	Fertilization cost/RMB	Spraying cost/RMB	Total cost/RMB
T1	766.67	175.00	437.50	262.50	1641.67
T2	516.67	555.56	375.00	350.00	1797.22
T3	766.67	1000.00	375.00	350.00	2491.67

*Based on 66.7 thousand m^2^. **Including all labor costs for irrigation during the rice cultivation process.

In soil preparation stage, it was mainly because T2 did not require 100% irrigation, but only about 60% of the irrigation. As a result, T2 area has a 32.6% reduction in labor costs compared to T1 and T3 areas. T2 and T3 had high loads of ground equipment, which consequently led to 16.5% higher labor cost for fertilizer application in T1 than T2 and T3. Pesticide spraying by mUAV (T1) had the advantage of reducing labor costs by 25% in T1 area compared to the other two areas. The UAV had an advantage over the rice direct seeder, but the advantage was not significant with higher only 8.7%. Compared to transplanting, the mUAV significantly reduced labor costs by 34.1%.

## Discussion

With the increasing application of UAVs in precision agriculture, the CFD was applied to the improve the downwash airflow of agricultural UAVs, from which the particle motion in the airflow was analyzed. For example, Yang et al. (2017, 2018a) simulated the velocity distribution of the downwash airflow field and the spatial distribution of droplets during the hovering condition of multi-rotor aircraft based on the Fluent k-ε turbulence model, which was verified by combining with indoor hovering experiments. From the wind field simulation results, it reflected the overall wind field spatial distribution pattern, and the simulated value of the average wind speed at the marker point was within 9% error with the experimental measurement results.

In this study, we simulated the wind field of the designed mUAV to provide a theoretical basis for the parameter setting of the field test. The different flow velocity of the downwash airflow from the center outward caused the effect of the flow field spreading outward from top to bottom. This resulted in an increase in spray width, and the spray width was proportional to the flight height (Yang et al., 2018b). The airflow along the outer rotor caused two peaks in a certain range. The effect became relatively poor (Zhang et al., 2016) when the multi-rotor UAV was operated at an altitude below 1 m. In addition, when the wind speed was smaller horizontally and larger (Chen et al., 2017a) vertically downward, the rotor’s downwash airflow had better deposition uniformity. Particles in the air were mainly affected by wind field forces and gravity, etc. When out of the downwash airflow region, they were mainly affected by the environmental wind speed, which was non-constant, and this affected the final position of the particles. These factors led to uneven distribution of rice seeds, fertilizer particles, and pesticide droplets. There was a risk of drift loss of pesticide droplets (Zhang et al., 2015; Wen et al., 2018). Therefore, based on the premise of satisfactory distribution uniformity and mUAV flight safety, the following operational parameters were selected in this work for experiments with a width of 4 m and a flight height 2−6 m at regular flight speed and lower flight height.

The simulation parameters were given as a guide, the specific operating parameters needed to be derived from actual tests. Song et al. (2018a) designed an air-powered UAV rice spreading device that used airflow to blow seeds out in different directions. The results showed that the UAV’s operating height in the range of 1−2.8 m had no significant effect on the spreading width and spreading uniformity. Considering the factors of spreading width and uniformity CV, as well as the field operating environment, the flight height of 2 m was considered to be the appropriate operation height of this UAV platform. Liu et al. (2020) studied the effect of quadrotor UAV spreading parameters on the seed distribution of *Astragalus membranaceus*. The results showed that the flight height was the most important factor affecting the uniformity of seed distribution. Meanwhile, the height of 1.5 m was recommended as the optimal operation height. Wang et al. (2016a,b) analyzed the distribution characteristics of droplets in different parts of space by comparing different UAVs, different flight modes, flight speeds, flight heights, and crosswinds using a spatial mass balance test. The results showed that the wind speed was fast and strong when the UAV flew at 2 m height. As the height increased, the airflow in the vertical direction weakened significantly. Although the increased flight altitude reduced the dispersion of droplet deposition rate and improved the uniformity of droplet distribution, there was a significant upward trend in the percentage of downwind drift. The above results showed that it was a double-edged sword that the method of adjustment of distribution uniformity by flight height. The airflow motion gradually decreased with increase of height, and there was a gradual diffusion change from vertical downward motion to horizontal motion (Shi, 2015). Simply increasing the flight height had an effect on the uniformity of distribution. However, as the flight height increased, the particles were weakened by the force of the downwash airflow, and it was susceptible to interference from ambient wind speed. Therefore, the operating parameters chosen in this study were flight height of about 2.5 m for broadcast application, and height of 3 m for pesticide application.

The application of UAVs in the whole process of rice cultivation or in individual segments can be seen to satisfy the requirements of modern paddy cultivation. Li et al. (2016) used a small multi-rotor UAV for rice broadcasting, the results showed that CV was far smaller than that of artificial broadcasting. The average yield of field broadcasted by UAV was 7,705.5 kg/hm^2^, implying that rice air broadcasting by UAV was feasible. Zhu et al. (2021) conducted a comparative trial of five cultivation methods: mechanical transplanting, unmanned machine seeding, mechanical precision hole sowing, mechanical seedling throwing, and manual seeding. The results showed that the number of seedlings in descending order was manual seeding, UAV seeding, mechanical precision hole sowing, mechanical transplanting, and mechanical seedling throwing. The total effective number of spikes was higher in the treatments of unmanned seeding and mechanical transplanting. The theoretical yield of each treatment was in the order of mechanical precision hole sowing, mechanical seedling throwing, UAV seeding, mechanical transplanting, and manual seeding. The analysis of the labor cost in the seedling planting process showed that the mechanical precision hole sowing or UAV seeding method was worthy of promotion. Zheng et al. (2021) conducted a comparison test on four different seeding methods: mechanical powder seeding, precision hole direct seeding, UAV seeding, and manual seeding. The results showed that the highest number of seedlings and the highest effective spikes were achieved in the UAV seeding treatment, with the effective spikes reaching 3,811,500 spikes/hm^2^. The actual yield of UAV seeding was 6,549 kg/hm^2^, which was 1.2% lower than that of mechanical precision hole seeding. Moreover, the lowest labor cost was 40.5 Yuan/hm^2^ for the UAV broadcast. The results of our work showed similar trends, with the number of seedlings per square in descending order of mUAV direct seeder, mechanical rice direct seeder, and mechanical rice transplanter. The theoretical yields were in the downhill order of mechanical direct seeder, mUAV direct seeder, and mechanical transplanter.

With the strict emission limits for environmental protection regulations and limits for exhaust pollutions from diesel engines of non-road mobile machinery (GB20891, 2014), the UAV used electrical energy as power, compared to traditional agricultural machinery burning diesel to obtain power, which reduced pollutant emissions. The results of Xu et al. (2012) showed that the average fuel consumption for the whole process of rice production in the southern double-season rice area was 95.08 L/hm^2^, of which 36.65 and 37.88 L/hm^2^ was for tillage and harvesting, respectively, 12.15 L/hm^2^ for transplanting, and 8.4 L/hm^2^ for mechanized plant protection. Data from the National Bureau of Statistics of China (NBS, 2021) showed that it was 4,734 hectares in the sown area of early-season rice in 2021. It was only 42.26% of mechanical rice cultivation rate of China in 2015. In this study, it provided a viable solution for rice cultivation in the application of mUAV in seeding, fertilizing, and applying pesticides, which contributed tremendously to the reduction of labor commitment and mechanization enhancement.

In addition, the cost of farm machinery was different in the three segments of seeding, fertilization, and spraying. The total input cost of farm machinery used in the mUAV seeding, mechanical seeding, and mechanical transplanting was about 110 thousand RMB, 215 thousand RMB, and 235 thousand RMB. However, the tractor used in the mechanical direct seeder and transplanter test area can also be used in the tillage stage. Therefore, it reduced the cost of farm machinery inputs in mUAV pilot area with certain extent, and it was increased rate of farm machinery utilization, but it would also increase the rate of depreciation.

At present, China’s agricultural UAVs are mostly used in plant protection, UAV broadcast applications are still in the preliminary research and trial application stage. There are still many problems with UAV applications.

In the fertilization process, the mUAV had no advantage over ground fertilization machinery. Although the number of workers was reduced, the operating efficiency and labor costs were lower than those of ground machinery. This was mainly due to the weight limitation of mUAV, as traditional ground machinery can be loaded up to 800–1,000 kg. UAVs had great advantages over manual fertilization. The results of the study by Ren et al. (2021) showed that the efficiency of UAV fertilization was about 12.5 times higher than manual fertilization, and the cost of UAV fertilization was reduced to 18.45 RMB/hm^2^. Liu et al. (2019) designed a spreading device for granular herbicides, and the results showed that the control effect was not significantly different from manual application, and the operational productivity reached 80–120 667 m^2^/h, which was 15–25 times higher. Diao et al. (2020) compared it to mechanical precision hole seeding and UAV direct seeding trials. The results showed that the yield of both was comparable. Although the UAV was slightly lower than the mechanical precision hole seeding, the operational cost was reduced by about 50% and the operational efficiency was increased by more than 5 times. There are limitations in the UAV fertilizer application process, the same problems existed with ground machinery in the middle and late stages of rice growth and in complex hilly areas (Xie et al., 2013), such as poor adaptability, high operational intensity, and severe crushing of rice (Chen et al., 2012; Qi et al., 2016; Shi et al., 2018; Song et al., 2018b). Therefore, there is still a great market demand for mUAV fertilizer application.

In the soil preparation process, there were two main ways of mechanized rice direct seeding, water direct seeding, and dry direct seeding. Water direct seeding was primarily applied in the south, rice seeds was germinated working in a leveled soil without waterlogging. Dry direct seeding was mostly applied in the north, which can be directly in the sowing of seeds without germination, but it has higher requirements for the plot (Luo et al., 2019). At this stage, the mUAV pilot area had advantages over the water broadcast pilot area, with lower labor costs. However, mUAV had no advantage compared to dry broadcast.

After the rice emerged in the experiment, it was observed that the seedlings emerged unevenly at both ends of the field, which might be due to uneven sowing caused by the change in speed when the mUAV was changing rows and turning. As the same time, the shape of the centrifugal spreading was circular, which easily led to overseeding between adjacent widths and result in poor uniformity (Qin and Liu, 2006). Zhou et al. (2018) studied the effect of UAV spreading methods on the characteristics of rice plants in terms of lodging resistance. The results showed significant differences in the main physical characteristics of the stalks between the different spreading methods, which showed that the stalks were thin at the 2nd and 3rd internodes. Also, the plants were taller, with less folding resistance and a higher lodging index. Apart from that, conventional spreaders buried the seeds into the soil, but the seeds from the UAVs were exposed on the surface of the field which were vulnerable to the sun or being fed by birds, which affected the seeds’ rooting and germination. On the other hand, rice seeds must be germinated before spreading. If the turntable was rotated too fast, it hit the wall which caused damage to the seed buds. Maximum range was also limited, the continuous flight capacity of the battery was generally 10–20 min, which led to the efficiency of the UAV work that could not be fully developed. The field operation required to carry several batteries, which were high-cost. It is also a problem that the UAV industry currently confronts.

However, as of 2016 (Luo et al., 2019), China’s comprehensive rice mechanization level was 79.2%, with tillage, sowing, and harvesting levels of 99.3, 44.5, and 87.1%, respectively. This is only the achievement of moving from the primary stage to the intermediate stage, indicating that we still need to continue our efforts to completely solve the problem of mechanization of rice cultivation in China. China should learn from the advanced equipment and technology of developed countries to develop a proper route for itself, agricultural mechanization development of China cannot directly copy the way of other country, and agricultural mechanization of China can only be realized step by step (Yang et al., 2003).

## Conclusion and outlook

Conclusions: In this study, a mUAV with three functions of seeding, spreading fertilizer, and applying pesticide was developed. CFD numerical simulation was used to initially obtain downwash airflow characteristics, and feasible operating parameters were obtained through practical operation test methods in the field. Through the whole rice cultivation comparison test with mechanical direct seeder and mechanical transplanter, the mUAV was summarized and analyzed in terms of operational efficiency, labor input, and yield in the management process.

The main conclusions were drawn as follows. (1) The modular design of the mUAV, its products could be used in the three segments of rice seeding, pesticide application and fertilization, and its ability could meet the requirements of rice production. Compared with other mechanical cultivation methods, this mUAV operation method could reduce the input of machinery types. (2) The range of operations allowed for the mUAV was verified in numerical simulations and from the perspective of real measurements. There was a strong initial value of airflow directly below the rotor, and a height of more than 1.5 m is recommended. It was recommended to fly at a height of 2.5 m for seeding and 3 m for pesticide application. (3) A comprehensive comparison was conducted in the whole rice cultivation and the results showed that the efficiency of sowing seeds by mUAV was 2.2 times and 4 times higher than that of mechanical direct seeding and transplanting, respectively. The labor cost was reduced by 68.5 and 82.5%, respectively. The efficiency of mUAV application was 1.3 times higher than mechanical direct seeding and rice transplanting. The cost of labor was reduced by 25%. However, the mUAV fertilization was not as efficient as mechanical direct seeder and transplanter, with 80% lower operational efficiency and 14.3% higher labor costs.

Outlook: Although the development of UAV research and applications in China started slowly and initially relied heavily on state funding, several research institutions and universities have conducted research on agricultural UAV. Especially in recent years, China has been paying more and more attention to the development and research of agricultural UAV. By the end of 2015, more than 3,000 agricultural UAV had been put into agricultural production in China, the number of flight controllers had exceeded 2,500, and there were more than 400 manufacturing companies in related industries. As of 2020, China’s agricultural UAV holdings were about 100,000 units, with an additional demand of 50,000 units in 2020 alone, which showed that agricultural UAV were in a phase of rapid development. With the massive transfer of agricultural population, rural labor capacity was insufficient, which made the utilization of agricultural resources inefficient, and it even seriously affected the production efficiency of agriculture. In some areas, there were also problems such as desertion, which was not conducive to the rational optimization of the rural industrial structure. Especially in remote hilly mountainous areas, ordinary ground machinery could not work in the fields, and there was a great lack of machinery in the rice production process. The mUAV could be controlled remotely and could also automatically route its operations. This not only solved the difficult problem that some ground machinery hardly worked in the paddy field, but also did not cause damage to the rice. The emergence of the mUAV has become an important breakthrough in solving this problem.

## Data availability statement

The original contributions presented in this study are included in the article/supplementary material, further inquiries can be directed to the corresponding authors.

## Author contributions

PQ performed most of the experiments with the assistance of CW, LX, and XJ. PQ designed the study, analyzed the data, and wrote the manuscript with ZW, YZ, SW, and JM. PQ designed and improved the UAV with the help of BC and CL. YP helped PQ to manage the rice field. PQ, XH, and YL were involved in all the contents throughout. All authors contributed to the study conception and design, read, and approved the final manuscript.
